# New cofactors and inhibitors for a DNA-cleaving DNAzyme: superoxide anion and hydrogen peroxide mediated an oxidative cleavage process

**DOI:** 10.1038/s41598-017-00329-y

**Published:** 2017-03-23

**Authors:** Yanhong Sun, Rulong Ma, Shijin Wang, Guiying Li, Yongjie Sheng, Hongyue Rui, Jin Zhang, Jiacui Xu, Dazhi Jiang

**Affiliations:** 1 Key Lab for Molecular Enzymology & Engineering of the Ministry of Education, School of Life Sciences, Jilin University, 2699# Qianjin Street, Changchun, 130012 China; 2 College of Animal Sciences, Jilin University, 5333# Xi’an Road, Changchun, 130062 China

## Abstract

Herein, we investigated the effects of new cofactors and inhibitors on an oxidative cleavage of DNA catalysis, known as a pistol-like DNAzyme (PLDz), to discuss its catalytic mechanism. PLDz performed its catalytic activity in the presence of ascorbic acid (AA), in which Cu^2+^ promoted, whereas Fe^2+^ significantly inhibited the catalytic function. Since Fe^2+^/AA-generated hydroxyl radicals are efficient on DNA damage, implying that oxidative cleavage of PLDz had no relation with hydroxyl radical. Subsequently, we used Fe^2+^/H_2_O_2_ and Cu^2+^/H_2_O_2_ to identify the role of hydroxyl radicals in PLDz catalysis. Data showed that PLDz lost its activity with Fe^2+^/H_2_O_2_, but exhibited significant cleavage with Cu^2+^/H_2_O_2_. Because Fe^2+^/H_2_O_2_ and Cu^2+^/H_2_O_2_ are popular reagents to generate hydroxyl radicals and the latter also produces superoxide anions, we excluded the possibility that hydroxyl radical participated in oxidative cleavage and confirmed that superoxide anion was involved in PLDz catalysis. Moreover, pyrogallol, riboflavin and hypoxanthine/xanthine oxidase with superoxide anion and hydrogen peroxide generation also induced self-cleavage of PLDz, where catalase inhibited but superoxide dismutase promoted the catalysis, suggesting that hydrogen peroxide played an essential role in PLDz catalysis. Therefore, we proposed a catalytic mechanism of PLDz in which superoxide anion and hydrogen peroxide mediated an oxidative cleavage process.

## Introduction

In living systems, reactive oxygen species (ROS) include superoxide anion (O_2_^•−^), hydrogen peroxide (H_2_O_2_), hydroxyl radical (OH^•^), organic (lipid, alkyl, or short chain) hydroperoxides and hydroperoxide radicals (ROOH, ROO•), hypochlorous acid (HOCl), singlet oxygen (^1^O_2_), and ozone (O_3_)^1, 2^. Nowadays, it is widely accepted that ROS play a dual physiological role, not only in various diseases, but also in cellular homeostasis^3^. Among reactive oxygen species, hydroxyl radical is extremely reactive and able to attack many biomolecules, such as nucleic acids, proteins and lipids.

In biological studies, the major source of hydroxyl radicals comes from Fenton reaction of Fe^2+^ with H_2_O_2_. The Fenton reaction initiates the Equation 1, which is then followed by the indicated in Equations 2 and 3. The reaction termination is caused by the Equation 4. The Equations 2 and 3 were commonly known as the Haber-Weiss cycle^4^.

1 $${{\rm{Fe}}}^{2+}+{{\rm{H}}}_{2}{{\rm{O}}}_{2}\to {{\rm{Fe}}}^{3+}+{{\rm{HO}}}^{\mbox{--}}+{{\rm{HO}}}^{\bullet }$$

2 $${{\rm{HO}}}^{\bullet }+{{\rm{H}}}_{2}{{\rm{O}}}_{2}\to {{\rm{H}}}_{2}{\rm{O}}+{{{\rm{O}}}_{2}}^{\bullet \mbox{--}}+{{\rm{H}}}^{+}$$

3 $${{{\rm{O}}}_{2}}^{\bullet \mbox{--}}+{{\rm{H}}}^{+}+{{\rm{H}}}_{2}{{\rm{O}}}_{2}\to {{\rm{O}}}_{2}+{{\rm{HO}}}^{\bullet }+{{\rm{H}}}_{2}{\rm{O}}$$

4 $${{\rm{Fe}}}^{2+}+{{\rm{HO}}}^{\bullet }+{{\rm{H}}}^{+}\to {{\rm{Fe}}}^{3+}+{{\rm{H}}}_{2}{\rm{O}}$$

Fenton reagents have been expanded from the original Fe^2+^/H_2_O_2_ system to the Fe^2+^/H_2_O_2_/AA (Fe^2+^/AA) system by introducing ascorbic acid (AA)^5, 6^. Meantime, it was reported that Cu^2+^ could also induce Fenton-like reactions into the Cu^2+^/AA (Cu^2+^/H_2_O_2_) system. In some studies, researchers have found that the Cu^2+^/AA (Cu^2+^/H_2_O_2_) system leads to DNA cleavage and damage^7, 8^. The cause has been attributed to the production of hydroxyl radicals by Fenton reaction (Eqs 5–8) to attack the deoxyribose DNA backbone and bases.

5 $${\rm{AA}}+2{{\rm{Cu}}}^{2+}\to {\rm{dehydroascorbic}}\,{\rm{acid}}+2{{\rm{Cu}}}^{+}+2{{\rm{H}}}^{+}$$

6 $$2{{\rm{Cu}}}^{+}+2{{\rm{O}}}_{2}\to 2{{\rm{Cu}}}^{2+}+2{{{\rm{O}}}_{2}}^{\bullet -}$$

7 $$2{{{\rm{O}}}_{2}}^{\bullet -}+2{{\rm{H}}}^{+}\to {{\rm{H}}}_{2}{{\rm{O}}}_{2}+{{\rm{O}}}_{2}$$

8 $${{\rm{Cu}}}^{+}+{{\rm{H}}}_{2}{{\rm{O}}}_{2}\to {{\rm{OH}}}^{\bullet }+{{\rm{OH}}}^{-}+{{\rm{Cu}}}^{2+}$$

In the mid-1990s, Carmi *et al.* using Cu^2+^/AA (or Cu^2+^) as cofactors obtained a series of oxidative cleavage DNA catalysis by *in vitro* selection, in which a pistol-like DNAzyme (PLDz) was the most active structure^9–11^. Based on its cofactors and structure, PLDz has been developed into Cu^2+^-, AA-, and glucose-biosensors, DNA molecular logic gates and a dual-catalytic allosteric DNAzyme^12–18^. However, little is known about the catalytic mechanism of PLDz except the oxidative cleavage of DNA. In this study, we found new cofactors for PLDz catalysis and further investigated their effects on the catalytic activity of PLDz. Our experimental data excluded that PLDz catalyzed a hydroxyl radical-mediated cleavage reaction and supported that superoxide anion and hydrogen peroxide might play a critical role in the oxidative cleavage process.

## Results

### General cofactors requirements for PLDz function

**Figure 1:**
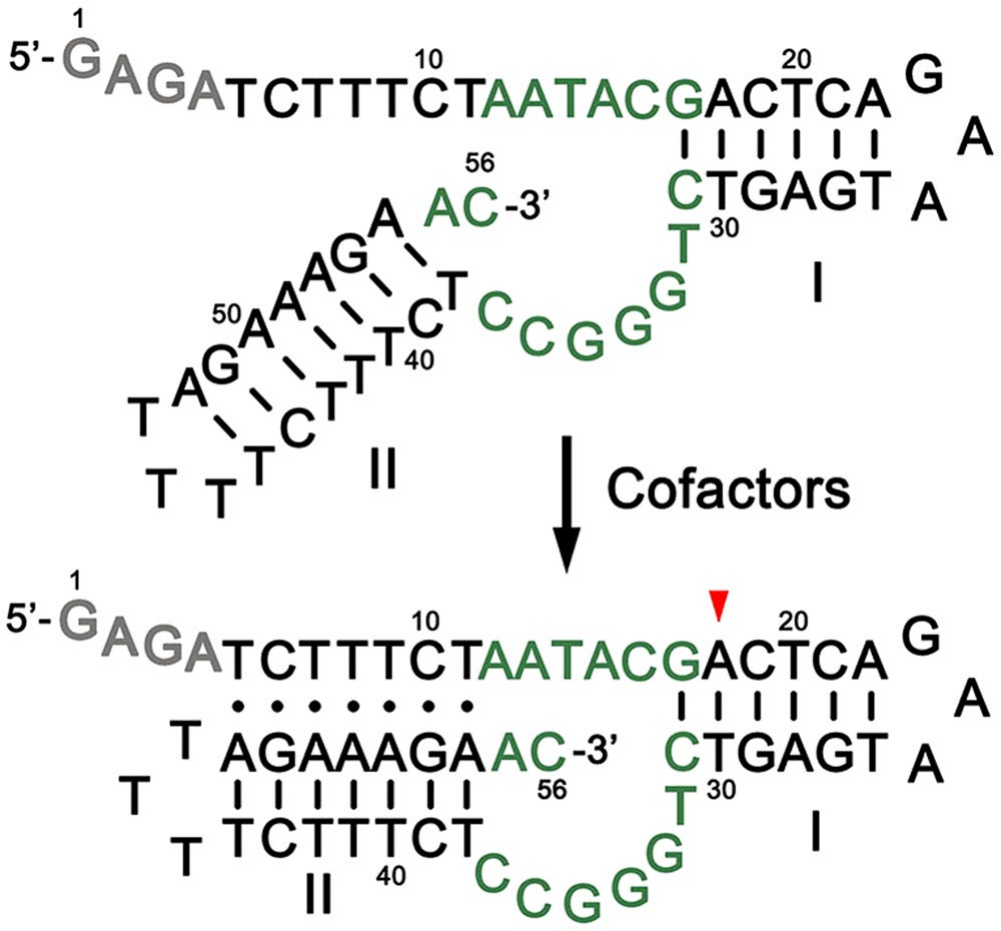
The sequence and secondary structure of a *cis* pistol-like DNAzyme. The green letters correspond to the conserved nucleotides of catalytic core. The red arrowhead indicates the major site of DNA cleavage. I and II designate stem-loop structures, where lines indicate Watson-Crick base pairs and dots represent triplex interactions.

A 56-nucleotide version of *cis* pistol-like DNAzyme (PLDz) was shown in Fig. 1. PLDz composes of a 15-nucleotide active core surrounded by a triple helix in the left arm and a double stranded helix in the right arm. The addition of GAGA at 5′ end allows separation of cleavage fragments by denaturing gel electrophoresis.

**Figure 2:**
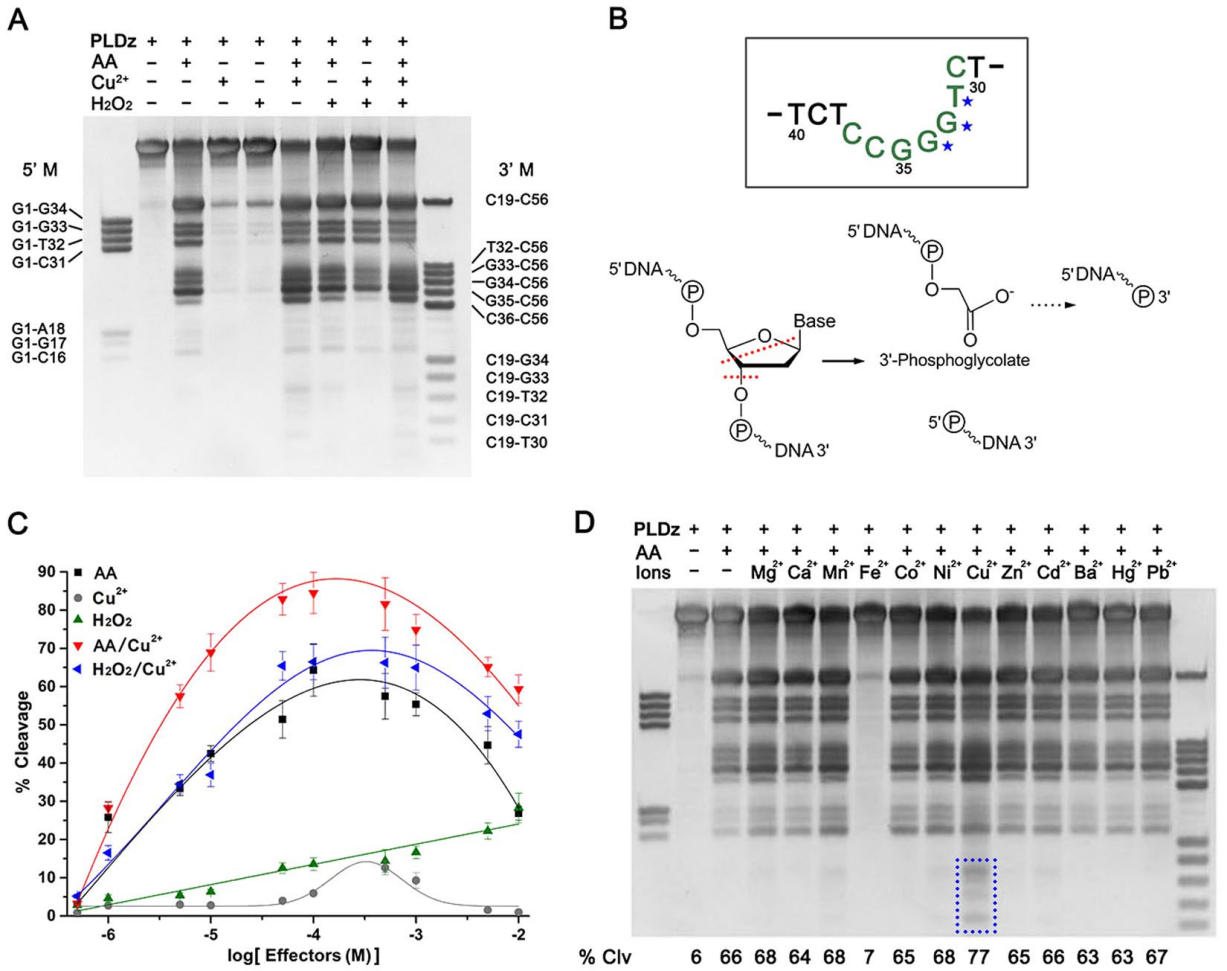
PLDz catalysis in the presence of different cofactors. (**A**) Effects of general cofactors on PLDz-catalyzed cleavage. PLDz (1 μM) was incubated with 100 μM cofactors at 23 °C for 2 hr in a mixture containing 50 mM Tris-HCl (7.0) and 300 mM NaCl. Lanes designated 5′ M and 3′ M were synthetic DNAs of different lengths as indicated, each with a sequence that corresponds to the 5′ and 3′ terminus (including middle fragments) of the PLDz, respectively (Supplementary Table 1). Letter-numbers indicate the lengths of these marker DNAs. Reaction products were separated by denaturing (7 M urea) 20% PAGE and were visualized by GelRed dye staining. (**B**) Oxidative cleavage of the target site deoxynucleotide. The DNA in the grey square represents the part structure of PLDz. The blue asterisks indicate the minor sites of DNA cleavage. The red dashed lines depict the possible locations of deoxynucleoside cleavage fragmentation. Dashed arrow indicates the possible conversion of phosphoglycolate into phosphate. (**C**) Concentration-dependent induction of PLDz function by cofactors. (**D**) Analysis of the function of PLDz with divalent metal ions in the presence of AA. The dashed box represents small cleavage fragments of PLDz including c19-c31, c19-t32 and c19-g33 (Supplementary Figure 1), which appeared more clearly with increased levels of exposure (Supplementary Figure 2) Reactions were conducted as described in part A. Note that only Fe^2+^ inhibited PLDz activity. Cropped gels are used in Fig. 2A and D, their full-length gels are presented in Supplementary Figure 3.

In previous studies, PLDz was generally labeled at the 5′ end with [γ-^*32*^P] ATP by T4 polynucleotide kinase^9–11, 18^. Using 5′ end-labeled PLDz for analysis, only 5′ cleavage fragments can be observed by autoradiography, while 3′ and middle cleavage fragments can′t be identified. In here, we used label-free PLDz for analysis. All cleavage products including 5′, 3′ and middle cleavage fragments can be separated by gel electrophoresis and detected with GelRed staining. As shown in Fig. 2A, catalysis by PLDz with Cu^2+^ or H_2_O_2_ alone has only been observed with low efficiency, which is consistent with previously reported data^10, 11, 18^. Surprisingly, AA as the sole cofactor was able to efficiently support cleavage of PLDz. In addition, PLDz showed slightly better catalytic activity in the Cu^2+^/AA system as compared to the Cu^2+^/H_2_O_2_ system. And addition of H_2_O_2_ did not improve cleavage efficiency compared with the corresponding Cu^2+^/AA. In Fig. 2A, self-cleavage of PLDz can generate multiple fragments, indicating that there exist several cleavage sites. These multiple cleavage sites could be predicted by comparison of DNA fragments lengths with 5′ and 3′ marker DNAs (Supplementary Figure 1). The a1 and a2 bands corresponded to the 5′ and 3′ cleavage fragments occurred at the cleavage site A18, which has been reported as the major cleavage site^10, 11^. The b1–3 and b4–7 bands could lead to the 5′ and 3′ cleavage fragments occurred at the minor cleavage sites. The c1–3 bands could be small cleavage fragments cleaving simultaneously at both major and minor cleavage sites. As a result of b1–7 and c1–3 bands, the minor cleavage sites in PLDz could be concluded as T32, G33 and G34. In Supplementary Figure 1, DNA cleavage fragments migrated differently with the same length of DNA markers, due to deoxynucleotide moieties bearing at their 3′ and 5′ ends. The newly generated 3-terminal end can be phosphoglycolate^11, 19, 20^ and might be further converted into 3′ phosphate^21^, while the 5′ end has a terminal phosphate group (Fig. 2B)^21^. In order to assess the influence of cofactors on PLDz activity, we conducted a series of DNAzyme assays using five groups of cofactors in a concentration series from 10 μM to 10 mM. As shown in Fig. 2C, PLDz showed a relation of linear dependence on H_2_O_2_ concentration, but exhibited bell-shaped dependence on other cofactors (Cu^2+^, AA, AA/Cu^2+^ and H_2_O_2_/Cu^2+^). Since a higher concentration of PLDz (1 μM) was used in our study, the optimal concentration of Cu^2+^ is 100 μM, ten times higher than concentration in previous reports^11, 18^.

In the following, we tested the activities of PLDz in the presence of AA and different metal ions (Mg^2+^, Ca^2+^, Mn^2+^, Fe^2+^, Co^2+^, Ni^2+^, Cu^2+^, Zn^2+^, Cd^2+^, Ba^2+^, Hg^2+^ and Pd^2+^) (Fig. 2D). At constant 100 μM AA, the equal concentration of Cu^2+^ was able to enhance PLDz catalysis. As shown in Fig. 2D, small cleavage fragments were observed upon addition of Cu^2+^and the cleavage yield was slightly higher than others. Similarly, PLDz only displayed a slight improved DNA cleavage activity in the presence of Cu^2+^, when incubated with different divalent metal ions alone (Supplementary Figure 4). Additionally, as shown in Fig. 2D, Fe^2+^ significantly inhibited the cleavage reaction of PLDz with AA as cofactor. PLDz lost its half catalytic activity by addition of 10 μM Fe^2+^ and almost abolished it at 100 μM Fe^2+^ (Supplementary Figure 5). In fact, the Fe^2+^/AA system generating hydroxyl radicals are efficient on DNA damage^22–25^. Therefore, we inferred that oxidative cleavage of PLDz had nothing to do with hydroxyl radicals.

### The role of hydroxyl radicals in the catalytic activity of PLDz

**Figure 3:**
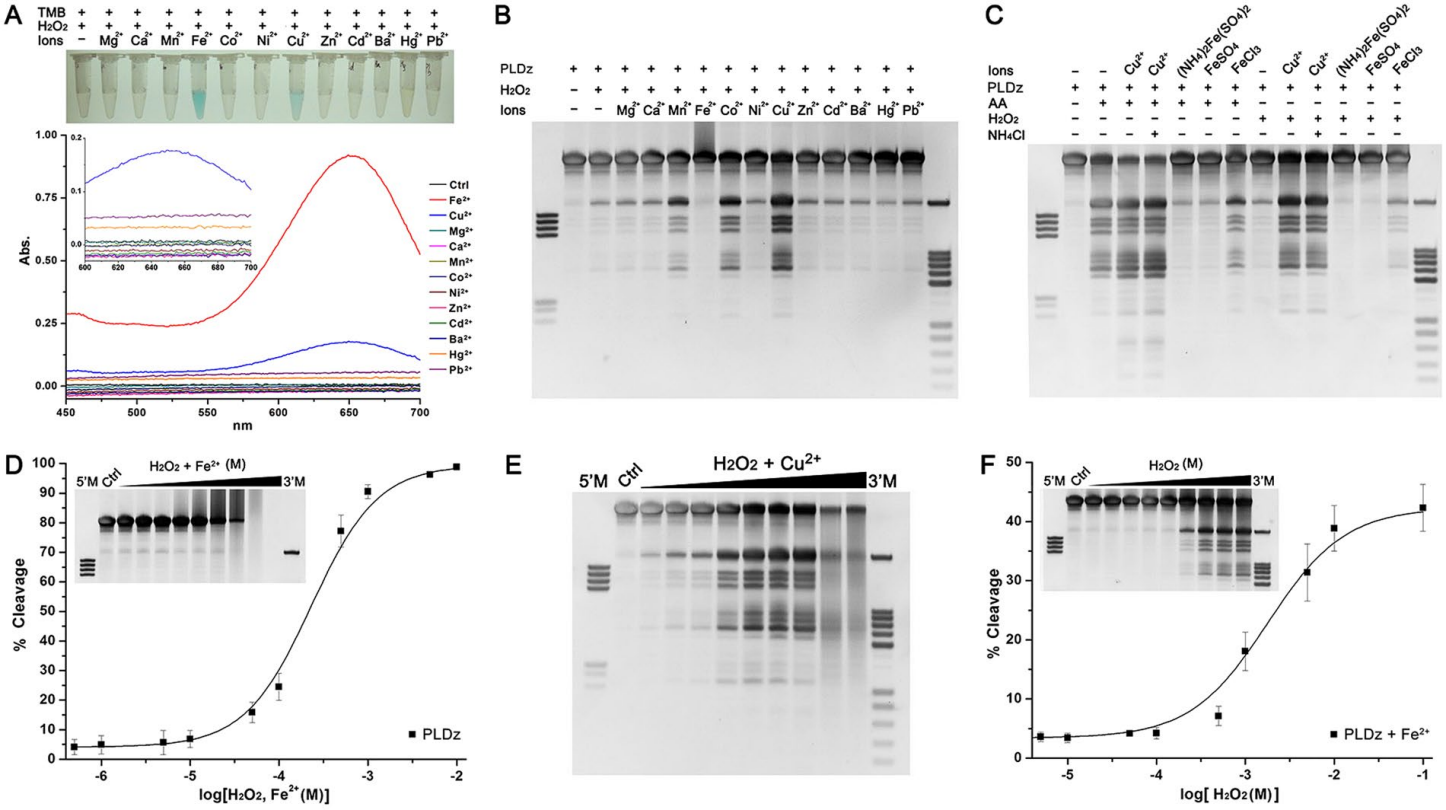
The role of hydroxyl radicals in the catalytic activity of PLDz. (**A**) Comparison of hydroxyl radical generation by treatment with different metal ions in the presence of H_2_O_2_. (**B**) Effects of divalent metal ions/H_2_O_2_ (100 μM) on the cleavage yield of PLDz. (**C**) Effects of Fe^2+^ (100 μM), Fe^3+^ (100 μM) and NH_4_^+^ (200 μM) on the cleavage yield of PLDz. (**D**) Effects of Fenton reagent (Fe^2+^/H_2_O_2_, 1 μM–0.01 M) on the cleavage yield of PLDz. (**E**) Effect of Cu^2+^/H_2_O_2_ concentration (1 μM–0.01 M) on the cleavage yield of PLDz. (**F**) Effect of H_2_O_2_ concentration (10 μM–0.1 M) on the cleavage yield of PLDz in present of 100 μM Fe^2+^. Reaction condition: 300 mM NaCl and 50 mM Tris-HCl (pH 7.0) at 23 °C for 2 hr. The error bars represented the standard deviations from three repeated measurements. Cropped gels are used in Fig. 3B–F, their full-length gels are presented in Supplementary Figure 8.

To further investigate the role of hydroxyl radicals in the catalytic activity of PLDz, we first evaluated the effects of different divalent metal species (Mg^2+^, Ca^2+^, Mn^2+^, Fe^2+^, Co^2+^, Ni^2+^, Cu^2+^, Zn^2+^, Cd^2+^, Ba^2+^, Hg^2+^ and Pd^2+^) on hydroxyl radical generation in the presence of H_2_O_2_ as measured by 3,3′,5,5′-tetramethylbenzidine (TMB) oxidation. Apparently, Fe^2+^/H_2_O_2_ can produce a more amount of hydroxyl radicals comparing with Cu^2+^/H_2_O_2_ (Fig. 3A). In addition, no hydroxyl radical was produced by treatment with other metal ions/H_2_O_2_. The hydroxyl radical formation rate from Fe^2+^/H_2_O_2_ was very high and its initial point had already reached its maximum peak (Supplementary Figure 6). In contrast, the hydroxyl radicals produced in Cu^2+^/H_2_O_2_ system grew linearly over the time.

We analyzed the different effects of metal ions/H_2_O_2_ on the cleavage reaction of PLDz (Fig. 3B). PLDz exhibited enhanced activities when treated separately with Cu^2+^, Co^2+^ and Mn^2+^ in the presence of H_2_O_2_, in the order Cu^2+^ > Co^2+^ > Mn^2+^. Other metal ions (Mg^2+^, Ca^2+^, Ni^2+^, Zn^2+^, Cd^2+^, Ba2^+^, Hg^2+^ and Pd^2+^) had no effect and Fe^2+^ showed a significant inhibitory effect. To further confirm the inhibitory effect on PLDz catalysis is due to Fe^2+^ rather than NH_4_^+^ in (NH_4_)_2_Fe(SO_4_)_2_ or Fe^3+^, we also investigated the effects of FeSO_4_, FeCl_3_ and NH_4_Cl on PLDz catalysis. Results showed that (NH_4_)_2_Fe(SO_4_)_2_ and FeSO_4_ inhibited the cleavage of PLDz, but FeCl_3_ and NH_4_Cl did not lower PLDz catalysis in the presence of AA or H_2_O_2_ (Fig. 3C). These results indicated that Fe^2+^ but not Fe^3+^ had an inhibitory effect on PLDz catalysis, while NH_4_^+^ in (NH_4_)_2_Fe(SO_4_)_2_ did not interfere with self-cleavage of PLDz.

So far, many papers have reported that hydroxyl radicals produced by Fe^2+^/H_2_O_2_ possess the ability to significantly destroy the DNA structure^26–28^. Our observation on DNA damage by Fe^2+^/H_2_O_2_ (Fig. 3D) is in agreement with previous reports. At 100 μM Fe^2+^/H_2_O_2_, there was no site-specific cleavage fragment but a smeared DNA observed, indicating that DNA damage occurred. At 1 mM Fe^2+^/H_2_O_2_, obvious smeared bands were displayed on the gel; at 5 mM Fe^2+^/H_2_O_2_, DNA was significantly damaged and could not bind the DNA staining reagent, showing a significant smear on the gel. At 10 mM Fe^2+^/H_2_O_2_, DNA was completely damaged and nothing appeared on the gel. This result provided a proof that hydroxyl radicals did not play a role in catalysis of PLDz. In Fig. 3D, Fe^2+^ resulted in an upper diffusion band, because a brown precipitate was formed by oxidation of Fe^2+^ to Fe^3+^ (Supplementary Figure 7) and decreased DNA electrophoretic mobility.

Both Fe^2+^ and Cu^2+^ can generate hydroxyl radicals in the presence of H_2_O_2_ (Fig. 3A and Supplementary Figure 6), however, they performed the opposing effects on PLDz catalysis (Fig. 3B and C). The rapid reaction of Fe^2+^ and H_2_O_2_ produces a large amount of hydroxyl radicals (Eq. 1), whereas the Cu^2+^/H_2_O_2_ system generates hydroxyl radicals also superoxide anions (Eq. 9 and 10)^29, 30^. Data (Fig. 3B and C) showed that Cu^2+^/H_2_O_2_ rather than Fe^2+^/H_2_O_2_ led to self-cleavage of PLDz, which indicated that superoxide anion took part in the cleavage process of PLDz. According to Eq. 10, high level of Cu^2+^/H_2_O_2_ (≥5 mM) can produce a large amount of hydroxyl radicals, which can non-specifically damage PLDz and leave smeared bands in gel, consistent with Fig. 3E. Other metal ions, Mn^2+^ and Co^2+^, were found to increase PLDz catalysis in the presence of H_2_O_2_ (Fig. 3B), suggesting that the Mn^2+^/H_2_O_2_ and Co^2+^/H_2_O_2_ systems can also generate superoxide anions.

9 $${{\rm{Cu}}}^{2+}+{{\rm{H}}}_{2}{{\rm{O}}}_{2}\to {{\rm{Cu}}}^{+}+2{{\rm{H}}}^{+}+{{{\rm{O}}}_{2}}^{\bullet \mbox{--}}$$

10 $${{\rm{Cu}}}^{+}+{{\rm{H}}}_{2}{{\rm{O}}}_{2}\to {{\rm{Cu}}}^{2+}+{{\rm{HO}}}^{\mbox{--}}+{{\rm{HO}}}^{\bullet }$$

At constant 100 μM Fe^2+^ and a range of H_2_O_2_ concentrations from 10 μM to 100 mM, we found that the activity of PLDz was recovered at the concentrations of H_2_O_2_ above 5 mM (Fig. 3F). This result seems inconsistent with previous Fe^2+^/H_2_O_2_-induced unspecific DNA degradation shown in Fig. 3D. But in fact, hydroxyl radicals reacted with excess of H_2_O_2_ in Fe^2+^/H_2_O_2_ system via the Haber-Weiss reaction (Eq. 2) to form superoxide anions. Moreover, high level of H_2_O_2_ can also promote the cleavage of PLDz, Therefore, it was a clue that superoxide anion and hydrogen peroxide could play an essential role in the cleavage reaction of PLDz.

### Effects of pyrogallol and riboflavin on PLDz

**Figure 4:**
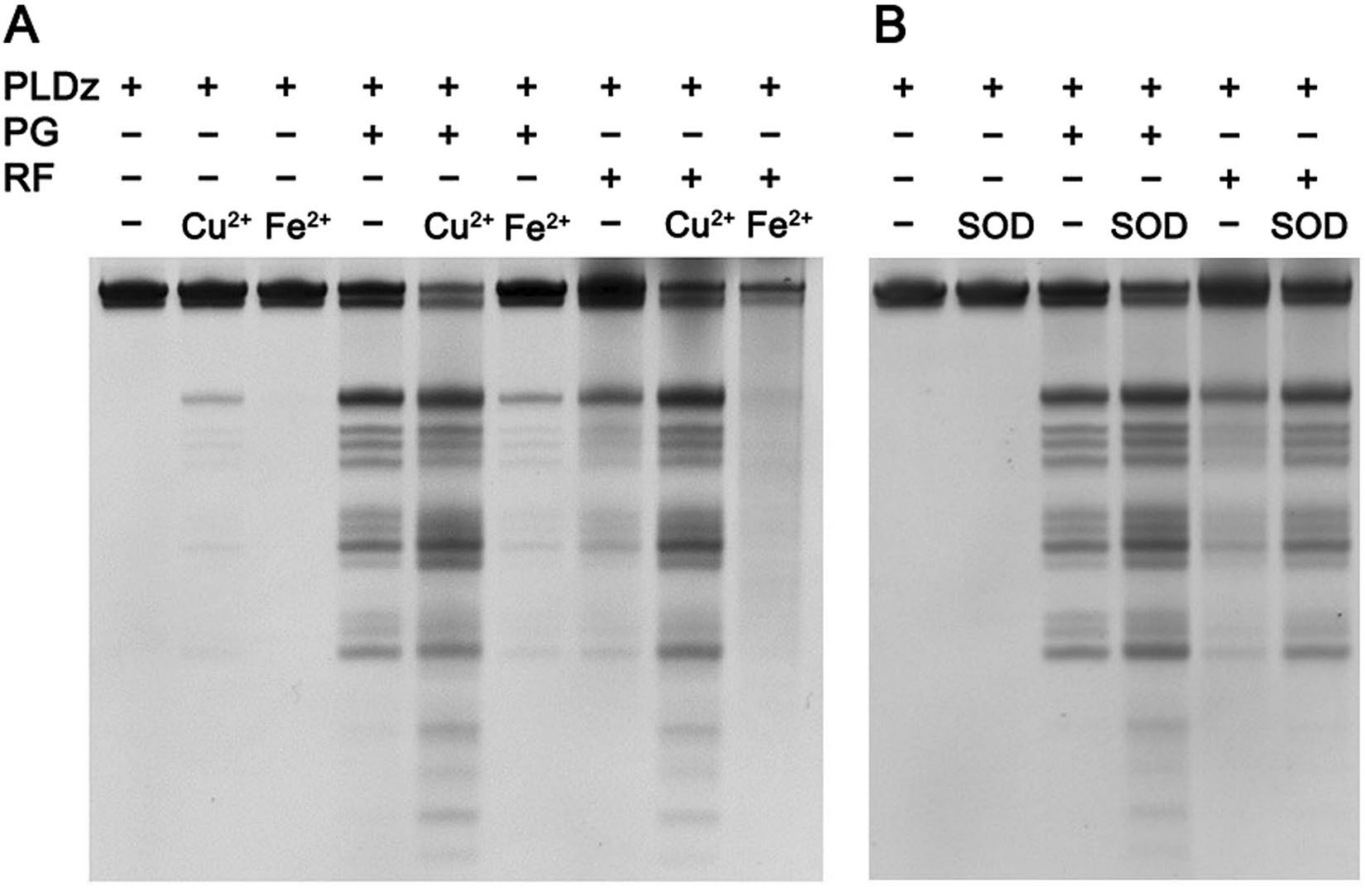
The function of pyrogallol (PG) and riboflavin (RF) on the catalytic activity of PLDz. (**A**) Effects of autoxidation of PG and photo-oxidation of RF on the cleavage reaction of PLDz. Reaction condition: 0.4 μM PLDz, 100 μM PG (RF), 100 μM Cu^2+^ (Fe^2+^), 300 mM NaCl and 50 mM Tris-HCl (pH 7.0) at 23 °C for 2 hr. The reaction system containing RF need to performed under a sunlight lamp. (**B**) Effects of SOD on the cleavage reaction of PLDz in the presence of H_2_O_2_. Reaction condition: 0.4 μM PLDz, 100 μM PG (RF), 0.3 U/μl SOD, 300 mM NaCl and 50 mM Tris-HCl (pH 7.0) at 23 °C for 2 hr. Cropped gels are used in Fig. 4A,B, their full-length gels are presented in Supplementary Figure 9.

Pyrogallol (PG) and riboflavin (RF) are generally utilized to generate superoxide anion and hydrogen peroxide by autoxidation and photo-oxidation, respectively^31, 32^. We chose both of them for further analysis. Results shown in Fig. 4A displayed that PLDz was self-cleaved when incubated with either pyrogallol or riboflavin with a concentration over 100 μM, suggesting that superoxide anion and hydrogen peroxide participated in PLDz-catalyzed DNA cleavage reaction. Addition of pyrogallol or riboflavin to 100 μM Cu^2+^ resulted in specific cleavage of PLDz in high yield. It may result from the superoxide anions generated through the reaction of Cu^2+^ and hydrogen peroxide. Fe^2+^ still inhibited the stimulatory effects of pyrogallol and riboflavin on PLDz catalysis, because Fe^2+^ not only participated in redox processes with pyrogallol and riboflavin but also used hydrogen peroxide to generate hydroxyl radical.

It is well known that superoxide dismutase (SOD) can efficiently convert superoxide anion to dioxygen and hydrogen peroxide (Eq. 11). We added SOD into the reaction system of pyrogallol or riboflavin to evaluate the effects of superoxide anion on PLDz catalysis. Results showed that PLDz performed an even stronger cleavage activity with the induction of pyrogallol/SOD (riboflavin/SOD) than pyrogallol (riboflavin) alone (Fig. 4B), indicating that hydrogen peroxide played an important role in pyrogallol- and riboflavin-mediated cleavage of PLDz. Similarly, hydrogen peroxide with the addition of SOD also improved the cleavage yield of PLDz (Supplementary Figure 10). This result is consistent with the earlier result about DNA cleavage mediated by H_2_O_2_/SOD by Han *et al.* ^33^.

11 $$2{{{\rm{O}}}_{2}}^{\bullet \mbox{--}}+2{{\rm{H}}}^{+}\to {{\rm{H}}}_{2}{{\rm{O}}}_{2}+{{\rm{O}}}_{2}$$

12 $$2{{\rm{H}}}_{2}{{\rm{O}}}_{2}\to 2{{\rm{H}}}_{2}{\rm{O}}+{{\rm{O}}}_{2}$$

**Figure 5:**
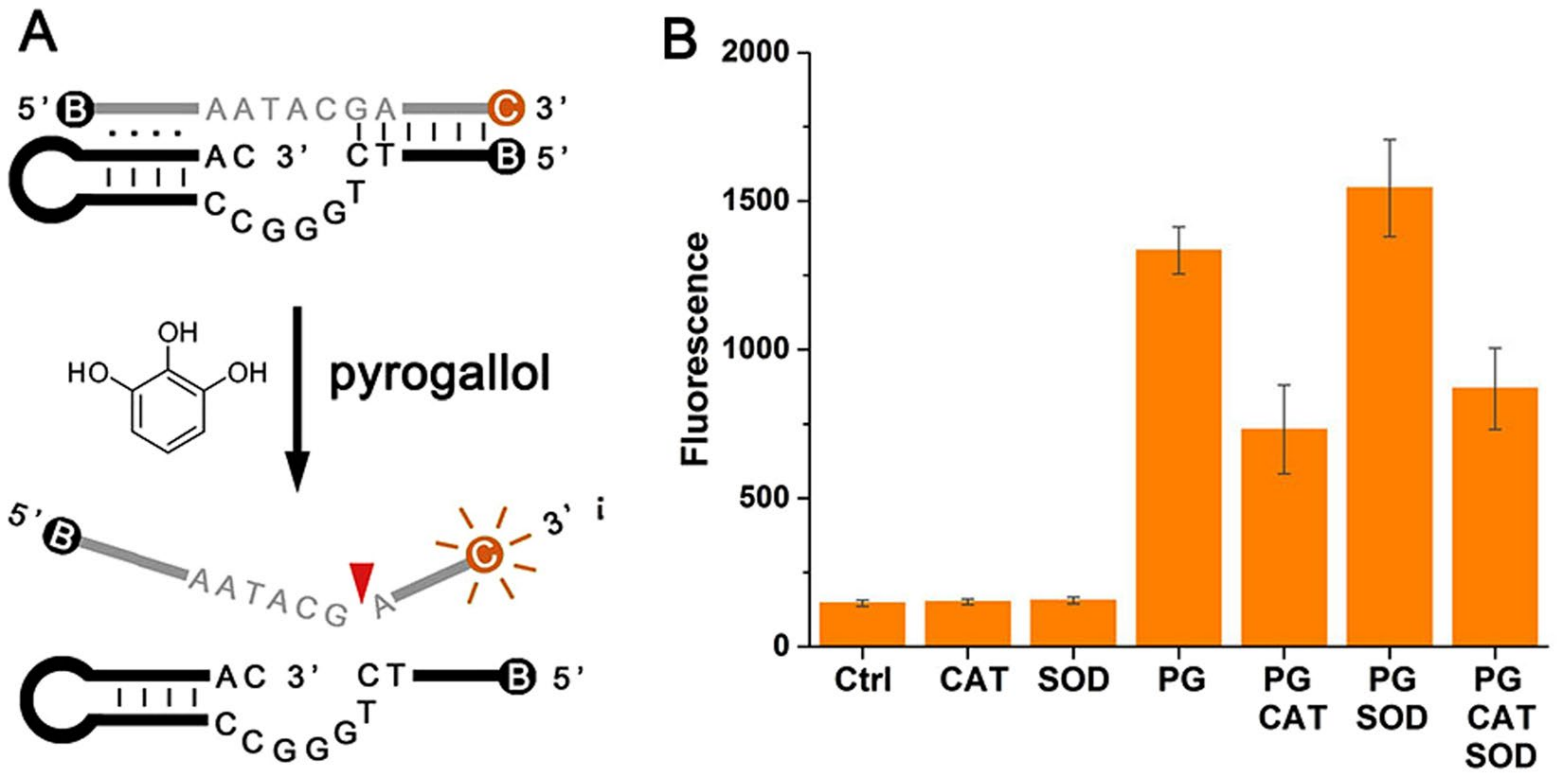
Effect of pyrogallol (PG) on a fluorescent labeled *trans*-PLDz. (**A**) Design of a fluorescent labeled *trans*-PLDz. C indicates Cy3, B indicates BHQ2. (**B**) Effects of CAT/SOD on the cleavage reaction of PLDz in the presence of PG. Reaction condition: 50 nM PL_B_, 50 nM S_BC_, 10 μM PG (50 mU/μl CAT, 5 mU/μl SOD), 300 mM NaCl and 50 mM MES (pH 6.0) at 37 °C for 1 hr.

In order to improve detection sensitivity, we designed a fluorescent labeled *trans*-PLDz (Fig. 5A), in which DNAzyme (PL_B_) contained 5′-quencher (BHQ2) and the substrate (S_BC_) was labeled with a Cy3 at the 3′-end and BHQ2 at the 5′-end. When PL_B_ bound S_BC_ forming an enzyme-substrate complex, Cy3 emission was quenched by the nearby BHQ2. In the presence of cofactors, PL_B_ catalyzed S_BC_ cleavage and the cleaved DNA fragments with Cy3 were released, resulting in increased fluorescence. As shown in Fig. 5B, the PL_B_ system emitted fluorescent signal in the presence of pyrogallol. The fluorescent intensity enhanced with the addition of SOD, which is in agreement with Fig. 4B. But it was dramatically reduced with the introduction of catalase (CAT) due to quick decomposition of hydrogen peroxide by CAT (Eq. 12) and inhibition of PL_B_ catalysis. These results suggested that SOD and CAT had opposite effects on PL_B_ and can be used as the positive and negative regulatory factors of PLDz to regulate its enzymatic activity. Besides, similar results in PL_B_ system were observed using riboflavin as cofactor (Supplementary Figure 11).

### Effects of hypoxanthine/xanthine oxidase on PLDz

**Figure 6:**
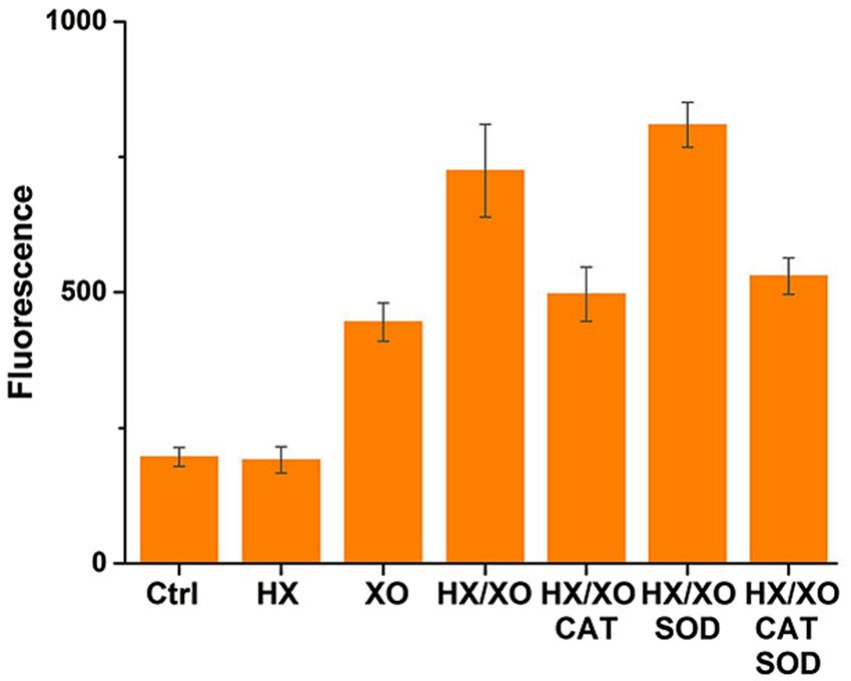
Effects of HX/XO on PLDz catalysis. Reaction condition: 50 nM PL_B_, 50 nM S_BC_, 10 μM HX, 1 mU/μl XO (50 mU/μl CAT, 5 mU/μl SOD), 300 mM NaCl and 50 mM MES (pH 6.0) at 37 °C for 1 hr.

We chose pyrogallol and riboflavin as generators of superoxide anion and hydrogen peroxide in previous text. Since the oxidation process of pyrogallol and riboflavin also produce other byproducts, we further used enzyme reactions to produce superoxide anion and hydrogen peroxide in order to decrease interference. Xanthine oxidase (XO) is generally recognized as a key enzyme in the catabolism of purines, which can catalyze hypoxanthine (HX) to generate uric acid, superoxide anion and hydrogen peroxide^34, 35^. In here, the hypoxanthine/xanthine oxidase system was utilized to produce superoxide anion and hydrogen peroxide. As shown in Fig. 6, hypoxanthine/xanthine oxidase led PL_B_ to cleave its DNA substrate. Fluorescent signal increased with the addition of SOD and decreased with additional CAT, consistent with the experimental results of pyrogallol and riboflavin.

Hydrogen peroxide played a key role in the process of PLDz cleavage according to the experimental results of hypoxanthine/xanthine oxidase system. Based on this principle, the oxidases that can generate hydrogen peroxide could also cause PLDz catalysis, such as amino-acid oxidase, glucose oxidase, gulonolactone oxidase, lysyl oxidase, monoamine oxidase, NAD(P)H oxidase, urate oxidase, and so on. In addition, we thought that some compounds similar to pyrogallol and riboflavin in structure can be used as the cofactors of PLDz, such as catechol, phloroglucinol, flavin mononucleotide (FMN), flavin adenine dinucleotide (FAD), etc.

## Conclusions

Our study indicates that pistol-like DNAzyme promoted an oxidative DNA cleavage using many cofactors and inhibitors. Newly discovered cofactors of PLDz include Mn^2+^/H_2_O_2_, Co^2+^/H_2_O_2_, pyrogallol(/Cu^2+^), riboflavin(/Cu^2+^), superoxide dismutase and hypoxanthine/xanthine oxidase, while its inhibitors include Fe^2+^ and catalase. The results that PLDz catalysis was assisted by Cu^2+^/H_2_O_2_ and inhibited by Fe^2+^/H_2_O_2_ excluded hydroxyl radical-mediated catalytic mechanism of PLDz. Data from the Cu^2+^/H_2_O_2_ system proved that superoxide anion played a critical role in the process of PLDz catalysis. Moreover, experimental results from pyrogallol, riboflavin and hypoxanthine/xanthine oxidase systems supported that hydrogen peroxide played an essential role in PLDz catalysis. Therefore, we proposed a catalytic mechanism of PLDz in which superoxide anion and hydrogen peroxide mediated an oxidative cleavage process.

## Methods

### Reagents and chemicals

The DNA oligonucleotides were purchased from Sangon Biotech Co., Ltd. (Shanghai, China) and purified by denaturing PAGE or HPLC (Supplementary Table 1). GelRed was purchased from Biotium Inc. Metal ions, cofactors and other reagents were of analytical reagent grade. Deionized and distilled water was used throughout the experiments.

### Detection of cleavage fragments of PLDz by polyacrylamide gel electrophoresis

DNA self-cleavage assays were conducted in 100 μl reaction solution containing 1 μM PLDz, 50 mM Tris-HCl (pH 7.0), 300 mM NaCl and cofactors. The reaction mixture was incubated at 37 °C for 2 hr and stopped by adding precipitants (200 μl (2× vol) 100% ethanol, 10 μl (1/10 vol) 3 M NaOAc (pH 5.2), 1 μl 10 mg/ml glycogen) for precipitation. The dried samples were dissolved in 20 μl loading buffer (4 M Urea, 10 mM EDTA, 25 mM Tris-HCl pH 7.5, 0.125‰ xylene cyanol FF, 0.125‰ bromophenol blue) and separated by electrophoresis in denatured 20% polyacrylamide gel. Gel was stained with GelRed dye for 10 min and visualized by UV transillumination. The cleavage yield was determined by the equation: cleavage (%) = all cleaved fragments/(non-cleaved fragments + all cleaved fragments) × %. Note: GelRed is a highly sensitive, low toxicity, fluorescent DNA stain designed to replace the highly toxic ethidium bromide (EtBr) and has similar staining protocol as EtBr. The post-staining protocol is to dilute GelRed 10,000× stock solution ~3300 fold to prepare a 3× working solution in 0.1 M NaCl. For example, 50 ml of the staining solution is composed of 15 μl GelRed 10,000× stock solution, 5 mL of 1 M NaCl, and 45 ml H_2_O.

### Visible spectroscopy detection

A 200 μl reaction mixture containing 200 μM metal ions, 1 mM H_2_O_2_, 0.1 mg/ml TMB and 50 mM Tris-HCl (pH 7.0). The reaction mixture was incubated at 23 °C for 3 min and scanned 450–750 nm by UV-2550 (Shimadzu), or measured the absorbance at 652 nm in the range of 0–300 sec.

### Fluorescence spectroscopy detection

A 100 μl reaction mixture containing 50 nM PL_B_, 50 nM S_BC_, 10 μM pyrogallol (10 μM riboflavin, 10 μM hypoxanthine/5 mU/μl xanthine oxidase), 50 mU/μl catalase, 5 mU/μl superoxide dismutase), 300 mM NaCl and 50 mM MES (pH 6.0) were incubated at 37 °C for 1 hr and scanned by Infinite F200 (Tecan). Parameter setting: Plate: Corning (3925), Excitation Wavelength: 530 nm and Emission Wavelength: 590 nm.

## Electronic supplementary material

supporting information
