# Supplementary material for: New cofactors and inhibitors for a DNA-cleaving DNAzyme: superoxide anion and hydrogen peroxide mediated an oxidative cleavage process
Source: Sci Rep. 2017 Mar 23;7:378. doi: 10.1038/s41598-017-00329-y (PMC5428237; doi:10.1038/s41598-017-00329-y)
Supplement: Supplementary file 1 — supporting information [file 41598_2017_329_MOESM1_ESM.docx]

**Supporting information**

New cofactors and inhibitors for a DNA-cleaving DNAzyme: superoxide anion and hydrogen peroxide mediated an oxidative cleavage process

Yanhong Sun^1,^*, Rulong Ma^1,^*, Shijin Wang^1^, Guiying Li^1^, Yongjie Sheng^1^, Hongyue Rui^1^, Jin Zhang^1^, Jiacui Xu^2^ & Dazhi Jiang^1^

^1^Key Lab for Molecular Enzymology & Engineering of the Ministry of Education, School of Life Sciences, Jilin University, 2699# Qianjin Street, Changchun 130012, China. ^2^College of Animal Sciences, Jilin University, 5333# Xi'an Road, Changchun 130062, China. *These authors contributed equally to this work. Correspondence and requests for materials should be addressed to D.J. (email: jiangdz@jlu.edu.cn) or J.X. (email: jcxu@jlu.edu.cn)

**Supplementary Table 1. Oligonucleotide sequences**

| Name | Sequences (5'→3') |
| --- | --- |
| PLDz | G_1_AGATCTTTC_10_TAATACGACT_20_CAGAATGAGT_30_CTGGGCCTCT_40_TTCTTTTAGA_50_AAGAAC_56_ |
| G1-G34 | G_1_AGATCTTTC_10_TAATACGACT_20_CAGAATGAGTC_31_T_32_G_33_G_34_ |
| G1-G33 | G_1_AGATCTTTC_10_TAATACGACT_20_CAGAATGAGTC_31_T_32_G_33_ |
| G1-T32 | G_1_AGATCTTTC_10_TAATACGACT_20_CAGAATGAGTC_31_T_32_ |
| G1-C31 | G_1_AGATCTTTC_10_TAATACGACT_20_CAGAATGAGTC_31_ |
| G1-A18 | G_1_AGATCTTTC_10_TAATAC_16_G_17_A_18_ |
| G1-G17 | G_1_AGATCTTTC_10_TAATAC_16_G_17_ |
| G1-C16 | G_1_AGATCTTTC_10_TAATAC_16_ |
| T32-C56 | T_32_G_33_G_34_G_35_C_36_CTCT_40_TTCTTTTAGA_50_AAGAAC_56_ |
| G33-C56 | T_32_G_33_G_34_G_35_C_36_CTCT_40_TTCTTTTAGA_50_AAGAAC_56_ |
| G34-C56 | T_32_G_33_G_34_G_35_C_36_CTCT_40_TTCTTTTAGA_50_AAGAAC_56_ |
| G35-C56 | T_32_G_33_G_34_G_35_C_36_CTCT_40_TTCTTTTAGA_50_AAGAAC_56_ |
| C36-C56 | T_32_G_33_G_34_G_35_C_36_CTCT_40_TTCTTTTAGA_50_AAGAAC_56_ |
| C19-G34 | C_19_T_20_CAGAATGAGT_30_C_31_T_32_G_33_G_34_ |
| C19-G33 | C_19_T_20_CAGAATGAGT_30_C_31_T_32_G_33_ |
| C19-T32 | C_19_T_20_CAGAATGAGT_30_C_31_T_32_ |
| C19-C31 | C_19_T_20_CAGAATGAGT_30_C_31_ |
| C19-T30 | C_19_T_20_CAGAATGAGT_30_ |
| PL_B_ | BHQ2-TGAGTCTGGGCCTCTTTCTTTTAGAAAGAAC |
| S_BC_ | BHQ2-TCTTTCTAATACGACTCA-Cy3 |

The green letters represent the conserved deoxyribonucleotides.


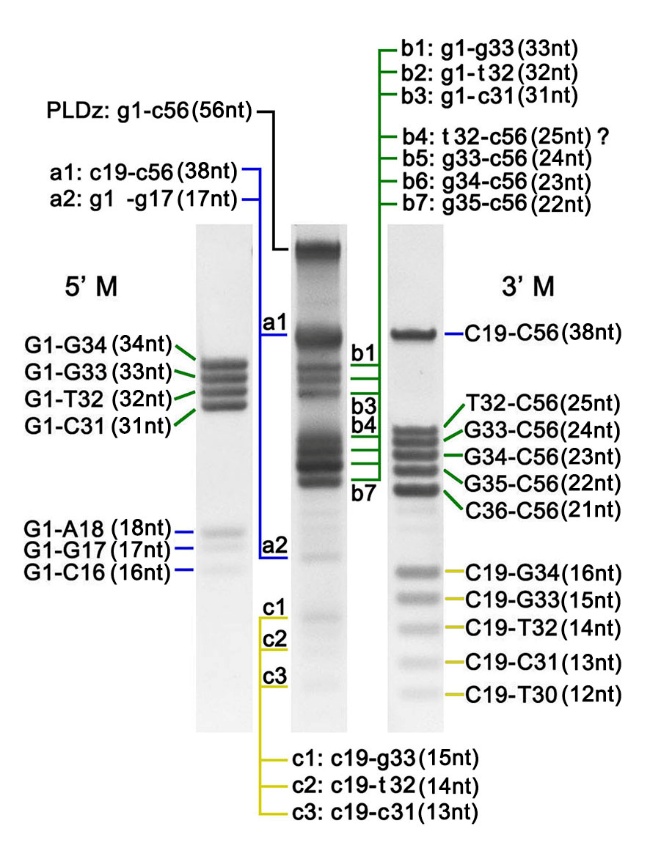


**Supplementary Figure 1. Analysis of the self-cleavage fragments of PLDz.** The predominant cleavage of PLDz occurred at the major site A18, resulting in two fragments a1 (3 'end cleavage product) and a2 (5' end cleavage product). Cleavage at predicted minor cleavage sites could generate multiple DNA fragments including b1-b7. Among them, the b1, b2 and b3 fragments could be 5’-terminal cleavage products corresponding to the cleavage sites at T32, G33 and G34 by comparison with 5' DNA Marker (5’ M). And the b5, b6 and b7 fragments could be 3’-terminal cleavage products corresponding to the cleavage sites at T32, G33 and G34 by comparison with 3’ DNA Marker (3’ M). The band b4 might be 3’-terminal cleavage fragment t32-c56 cleaved at the cleavage site C31, however there was no corresponding 5’-terminal cleavage fragment. Therefore, b4 needs to be further analyzed. Based on the fragments lengths, band c1, c2 and c3 could lead to the DNA fragments produced by simultaneous cleavage at both major and minor cleavage sites. In addition, DNA cleavage fragments migrated differently with the same length of DNA markers, due to the phosphoglycolate or phosphate groups at the 5’ and 3’ ends of these fragments.

**
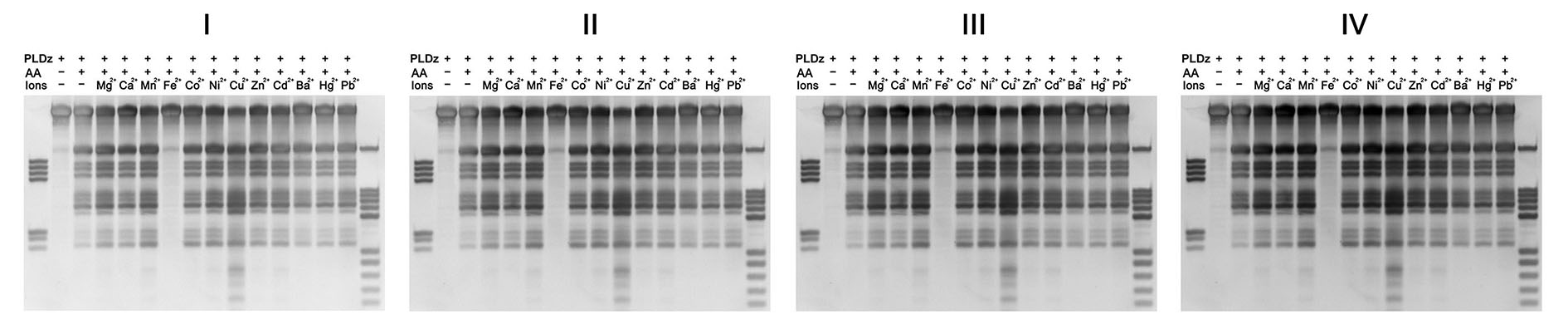
**

**Supplementary Figure 2. Small cleavage fragments of PLDz were presented with increased levels of exposure.** Exposure levels increased from left to right, II and Figure 2D were exposed at the same level. In the presence of Cu^2+^/AA, small cleavage fragments of PLDz (The dashed box shown in Figure 2D) appeared more clearly with increased exposure levels.

**
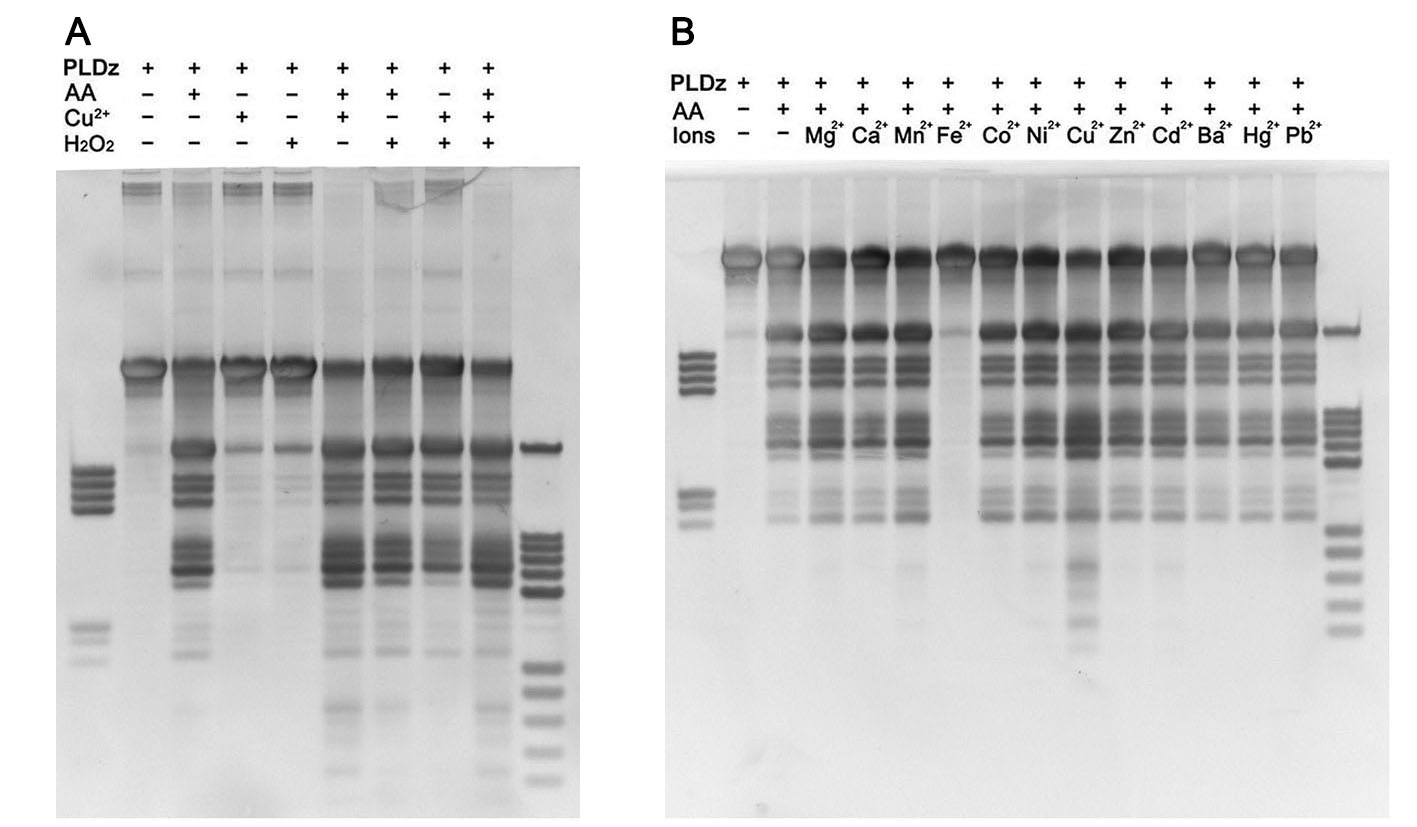
**

**Supplementary Figure 3. The full-length gels of Figure 2A and 2D.** (A) The full-length gel of Figure 2A. (B) The full-length gel of Figure 2D.


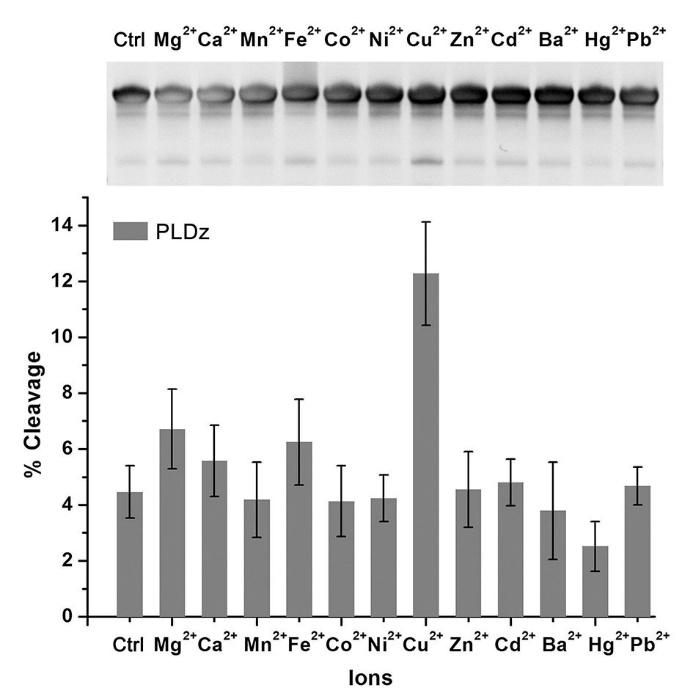


**Supplementary Figure 4. Differential effects of divalent metal ions on the cleavage yield of PLDz.** PLDz (1 μM) was incubated with 100 μM metal ions at 23 °C for 2 hr in a mixture containing 50 mM Tris-HCl (7.0) and 300 mM NaCl. The reaction products were separated by denaturing (7 M urea) 20 % PAGE and were visualized by staining with GelRed dye. The error bars represent standard deviations calculated from three parallel assays. Note that Cu^2+^ only slightly increased the cleavage yield of PLDz.


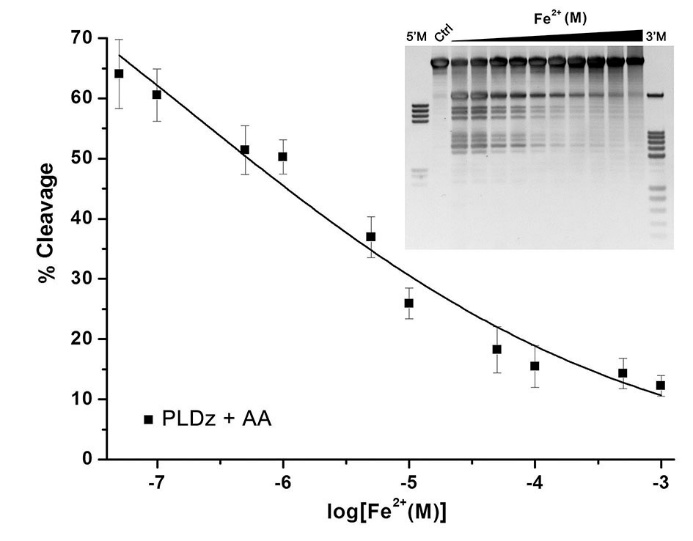


**Supplementary Figure 5. Inhibitory effect of Fe^2+^ on the cleavage yield of PLDz.** PLDz (1 μM) was incubated with Fe^2+^ (50 nM-1 mM) at 23 °C for 2 hr in a mixture containing 100 μM AA, 50 mM Tris-HCl (7.0) and 300 mM NaCl. The reaction products were separated by denaturing (7 M urea) 20 % PAGE and were visualized by staining with GelRed dye. The error bars represent standard deviations calculated from three parallel assays. Data showed that Fe^2+^ had more obvious inhibitory effect on PLDz catalysis with the increase of Fe^2+^.


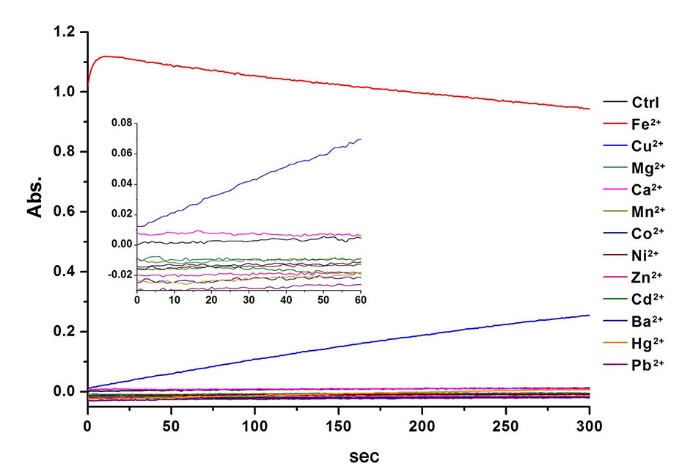


**Supplementary Figure 6. Hydroxyl radical generation induced by divalent metal ions/H_2_O_2_ over the time.** A 200 μl reaction mixture contained 200 μM metal ions, 1 mM H_2_O_2_, 0.1 mg/ml TMB and 50 mM Tris-HCl (pH 7.0). The reaction mixture was measured the absorbance at 652 nm in the range of 0-300 sec. Data showed that Fe^2+^/H_2_O_2_ system generated a large amount of hydroxyl radicals very fast; Cu^2+^/H_2_O_2_ system linearly produced hydroxyl radicals over the time; other metal ions/H_2_O_2_ system failed to produce hydroxyl radicals.


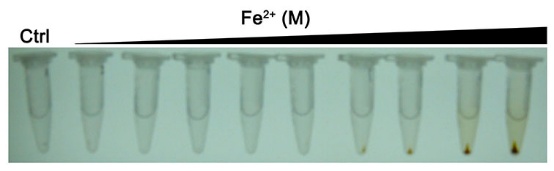


**Supplementary Figure 7. Reaction of Fe^2+^/H_2_O_2_ with PLDz.** PLDz (1 μM) was incubated with Fe^2+^/H_2_O_2_ (1 μM-0.01 M) at 23 °C for 2 hr in a mixture containing 50 mM Tris-HCl (7.0) and 300 mM NaCl and stopped by adding precipitants (200 μl (2 x vol) 100% ethanol, 10 μl (1/10 vol) 3 M NaOAc (pH 5.2), 1 μl 10 mg/ml glycogen) for precipitation. The brown precipitate was observed after centrifugation (14000 rpm, 15 min).

**
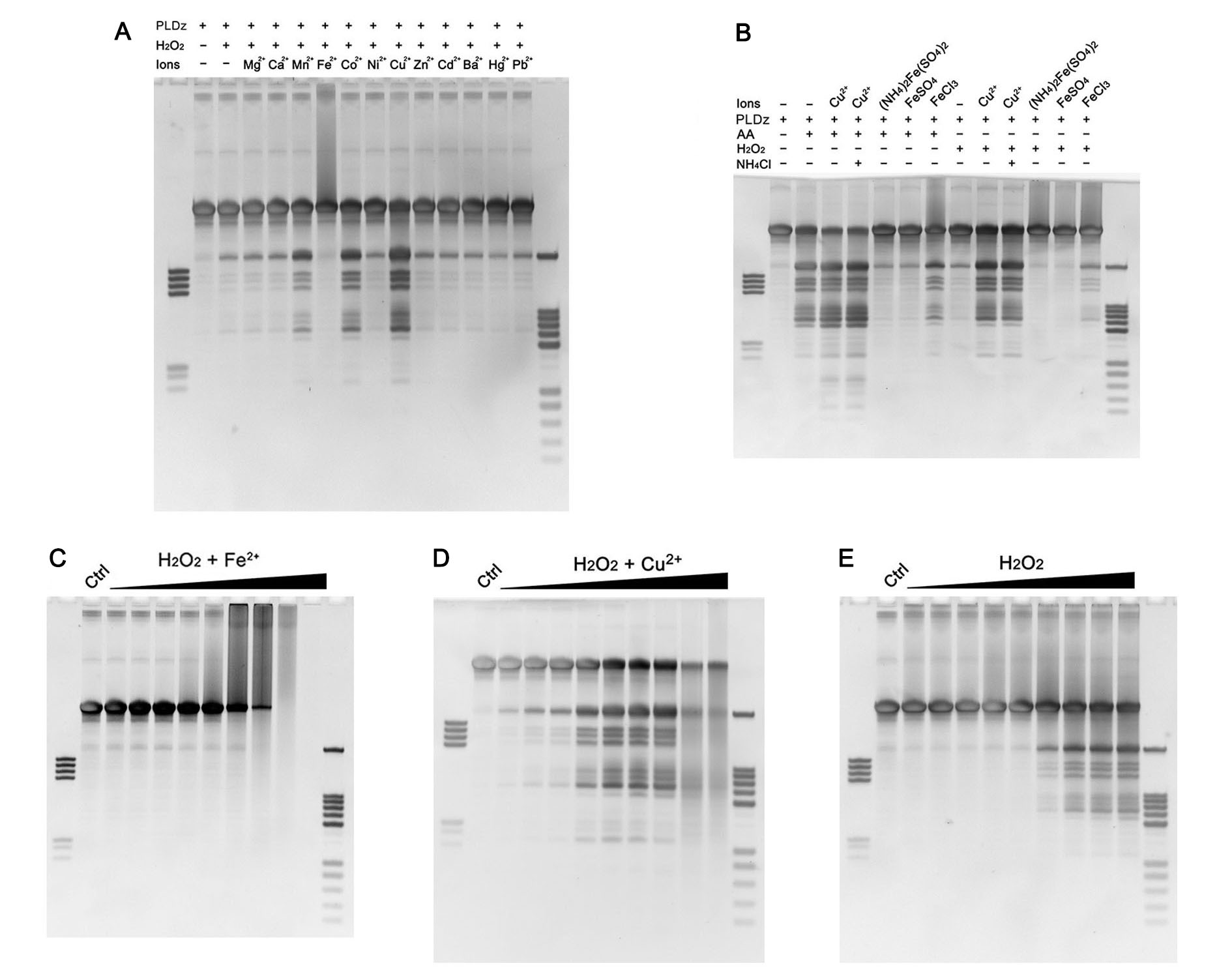
**

**Supplementary Figure 8. The full-length gels of Figure 3B, 3C, 3D, 3E and 3F.** (A) The full-length gel of Figure 3B. (B) The full-length gel of Figure 3C. (C) The full-length gel of Figure 3D. (D) The full-length gel of Figure 3E. (E) The full-length gel of Figure 3F.


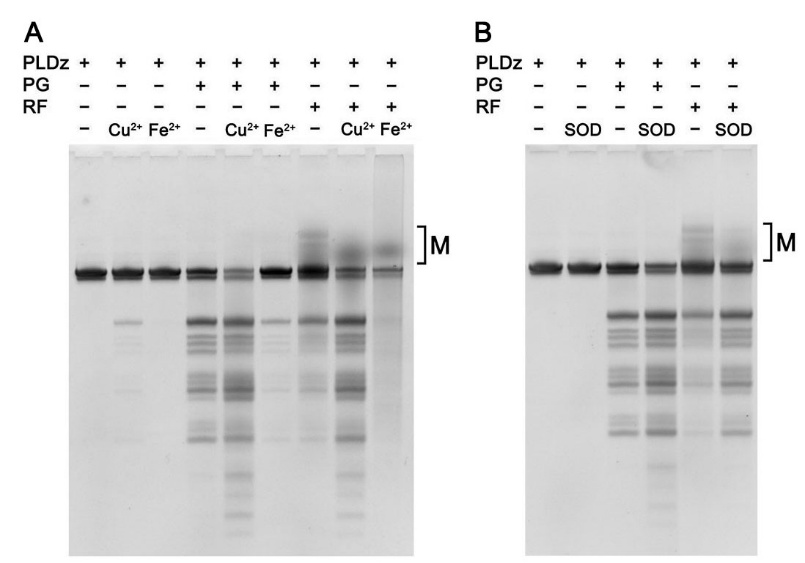


**Supplementary Figure 9. The full-length gels of Figure 4A and 4B.** (A) The full-length gel of Figure 4A. (B) The full-length gel of Figure 4B. M represents riboflavin, which was in green under UV, while DNAzyme stained with GelRed was in red. However, the gel was imaged as black-and-white color, which did not distinguish between green and red areas.


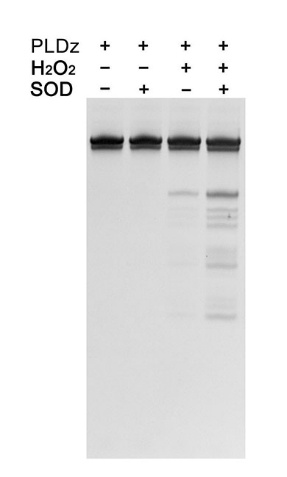


**Supplementary Figure 10. Effects of hydrogen peroxide and SOD on the cleavage reaction of PLDz.** Reaction condition: 0.4 μM PLDz, 100 μM H_2_O_2_, 0.3 U/μl SOD, 300 mM NaCl and 50 mM Tris-HCl (pH 7.0) at 23 °C for 2 hr. Data showed that SOD alone had no effect on PLDz, inclusion of SOD along with H_2_O_2_ enhanced the catalytic activity of PLDz when comparing with H_2_O_2_ treatment alone. These results are consistent with Figure 4B, indicating that SOD assisted the cofactor to promote the self-cleavage of PLDz.


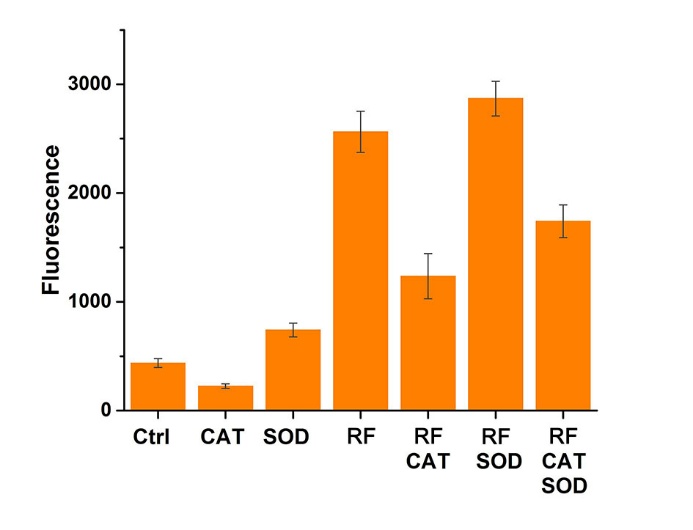


**Supplementary Figure 11. Effect of riboflavin (RF) on a fluorescent labeled *trans*-PLDz.** Reaction condition: 50 nM PL_B_, 50 nM S_BC_, 10 μM RF (50 mU/μl CAT, 5 mU/μl SOD), 300 mM NaCl and 50 mM MES (pH 6.0) at 37 °C for 1 hr. The reaction system containing RF needs to be performed under a sunlight lamp. Ctrl indicates no reaction. The error bars represent standard deviations calculated from three parallel assays. Data showed that the effect of RF on the self-cleavage of PLDz was enchanced by SOD and weakened by CAT, consistent with Figure 5.
